# The Sufferings of Christ

**Published:** 1887-09-03

**Authors:** 


### WORDS OF CONSOLATION.

[Communications for this column should be addressed, "Chaplain," care of the Editor of THE HOSPITAL, who will gratefully welcome any suggestions or inquiries from matrons, nurses, and the staff generally.]

### XLVIII.—THE SUFFERINGS OF CHRIST.

*(For Reading to the Sick.)*

HAVING briefly recalled to your mind the outline of our Blessed Lord's sufferings, we now invite you to consider more fully some of the details, beginning with Gethsemane. Where had He come from? He had not long left the Supper-table where He had eaten the Passover with His disciples, and ordained the Holy Sacrament of His Body and Blood with the words: "Take, eat; this is My Body which is given for you. Do this in remembrance of Me." The Agony was partly caused by foresight. He knew all things that should come upon Him. Can we doubt that upon going forth from the institution of this Holy Sacrament to endure the burden of sin He had on His mind the ingratitude of multitudes who would wound Him until His coming again by calling themselves Christians, and yet declining to obey His dying command? Ask yourself here whether you have not contributed by your neglect to this ever-accumulating denial of His heart's yearning He says to you with the same longing for your love to be returned as He expressed then, "With desire have I desired to eat this Passover with you" (LUKE xxii, 15). If, however, you are a faithful communicant you fulfil the great desire; you quench this thirst of your Redeemer's soul. It is well.

The upper room where Jesus had eaten the Passover with His disciples was close to the temple. So they had to wend their way through the streets of Jerusalem, until they found themselves outside the great walls of the city. He leads the way down the steep sides of the hill, across the brook Kidron, and up the opposite slope of the Mount of Olives. "To one who has visited the scene at the same season of the year, and at the same hour of the night; who has felt the solemn hush of the silence, even at this short distance from the city wall; who has seen the deep shadows flung by the ancient olive-trees, and the chequering of light that falls on the sward through their moonlight silvered leaves, it is more easy to realise the awe which crept over those few Galileans, as in almost unbroken silence, with something perhaps of secrecy, and with a weight of mysterious dread brooding over their spirits, they followed Him, who with bowed head and sorrowing heart walked before them to His willing doom" (Farrar). Far into the shade He went in more senses than one, to be overwhelmed and weighed down by some terrible revelations of the dark, hideous nature and consequences of sin. "Tarry ye here and watch with Me" (MATT. xxvi, 38). *(To be continued.)*

